# Efficient and gentle delivery of molecules into cells with different elasticity *via* Progressive Mechanoporation†

**DOI:** 10.1039/d0lc01224f

**Published:** 2021-05-12

**Authors:** Alena Uvizl, Ruchi Goswami, Shanil Durgeshkumar Gandhi, Martina Augsburg, Frank Buchholz, Jochen Guck, Jörg Mansfeld, Salvatore Girardo

**Affiliations:** Cell Cycle, Biotechnology Center, Technische Universität Dresden 01307 Dresden Germany; Max Planck Institute for the Science of Light & Max-Planck-Zentrum für Physik und Medizin 91058 Erlangen Germany salvatore.girardo@mpl.mpg.de; Medical Systems Biology, Medical Faculty and University Hospital Carl Gustav Carus, TU Dresden 01307 Dresden Germany; The Institute of Cancer Research London SW7 3RP UK jorg.mansfeld@icr.ac.uk

## Abstract

Intracellular delivery of cargo molecules such as membrane-impermeable proteins or drugs is crucial for cell treatment in biological and medical applications. Recently, microfluidic mechanoporation techniques have enabled transfection of previously inaccessible cells. These techniques create transient pores in the cell membrane by shear-induced or constriction contact-based rapid cell deformation. However, cells deform and recover differently from a given extent of shear stress or compression and it is unclear how the underlying mechanical properties affect the delivery efficiency of molecules into cells. In this study, we identify cell elasticity as a key mechanical determinant of delivery efficiency leading to the development of “progressive mechanoporation” (PM), a novel mechanoporation method that improves delivery efficiency into cells of different elasticity. PM is based on a multistage cell deformation, through a combination of hydrodynamic forces that pre-deform cells followed by their contact-based compression inside a PDMS-based device controlled by a pressure-based microfluidic controller. PM allows processing of small sample volumes (about 20 μL) with high-throughput (>10 000 cells per s), while controlling both operating pressure and flow rate for a reliable and reproducible cell treatment. We find that uptake of molecules of different sizes is correlated with cell elasticity whereby delivery efficiency of small and big molecules is favoured in more compliant and stiffer cells, respectively. A possible explanation for this opposite trend is a different size, number and lifetime of opened pores. Our data demonstrates that PM reliably and reproducibly delivers impermeable cargo of the size of small molecule inhibitors such as 4 kDa FITC-dextran with >90% efficiency into cells of different mechanical properties without affecting their viability and proliferation rates. Importantly, also much larger cargos such as a >190 kDa Cas9 protein–sgRNA complex are efficiently delivered high-lighting the biological, biomedical and clinical applicability of our findings.

## Abstract 2

Progressive Mechanoporation, a novel mechanoporation method that improves the delivery efficiency of molecules into cells of different elasticity *via* a multistage cell deformation controlled by a PDMS-based microfluidic platform.

## Graphical abstract

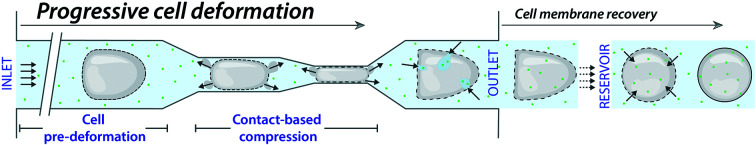

## Introduction

Cell membranes are essential for keeping cellular homeostasis by preventing entry of extracellular molecules into and out of cells. For a detailed understanding of cellular processes and possible therapeutic applications, it is crucial to deliver impermeable cargo molecules such as nucleic acids (*e.g.* small interfering RNAs or messenger RNAs), antibodies, nucleases or nanomaterials such as quantum dots and nanobodies across the cell membrane in an efficient and high-throughput fashion. Intracellular delivery, mostly the introduction of nucleic acids or proteins into living cells, is the mainstay of cell biology and increasingly also of clinical biology.^1^ Importantly, the intracellular delivery is an essential but yet often a limiting step in the clinic; for example, in the preparation of induced pluripotent stem cells for regenerative medicine^2–4^ and in the gene therapy based on *ex vivo* manipulation of patient cells with genome editing technologies such as CRISPR/Cas9.^5–7^

Among the available techniques for intracellular delivery,^1^ mechanoporation methods rely on mechanical forces to deform cells in suspension allowing for transient plasma membrane permeabilization in a highly effective, well-controlled and reproducible manner.^8,9^ Since these techniques do not require further modifications of molecules to be delivered, any external electric field effects or use of viral-vectors, all related biological toxicities and other limitations of electroporation as well as biochemical methods can be overcome.^1,8,9^ However, delivery efficiency of mechanoporation methods varies in different cell types.^10–12^ Further investigation of different cell parameters is needed to develop new strategies and enlarge the application of the methods. While a lot of attention has been dedicated to the applied forces and cell size, cell mechanical properties have been poorly investigated in relation to cell membrane poration.

A pioneering study employed a syringe loading technique to transiently permeabilize the cell membrane by directly applying shear forces through a hypodermic needle.^13^ Hallow *et al.* demonstrated, by simply flowing cells through microchannels, that shear-induced plasma membrane disruption led to intracellular uptake of the biomolecules. They found that the best delivery efficiency was obtained by applying high flow rates through small-diameter, short-length channels, suggesting that exposure to high shear stress for a short duration of time led to most optimal results. However, even though shear-induced intracellular delivery approaches are straightforward, they lack high loading efficiency and optimal cell viability.^14^

Higher delivery efficiency of different cargos in different cells, while keeping good cell viability, has been achieved by solid contact-mediated membrane permeabilization due to fast squeezing of cells in suspension through constrictions.^10,11,15–20^ Sharei *et al.* employed silicon-based microfluidic devices operated by an external pressure regulator to control the shear and compression rates experienced by cells for cytosolic diffusive delivery. This delivery method was dependent on cargo size due to size-dependent diffusive molecular loading, while different cell types under the same treatment showed different behaviour in uptake of the same or different cargos.^10^ Lam *et al.* enlarged the applicability of the same method to polydimethylsiloxane (PDMS)-based devices (cyto-PDMS) controlled by a syringe pump, making the device fabrication low-cost and accessible to rapid prototyping for the easy optimization in most academic research labs.^21^ However, their method relied on a multi-casting PDMS process and on the use of syringe pump-driven flow, with longer stabilization time compared to pressure-driven flow.^22^ This precludes stable and reproducible processing of small sample volumes. Furthermore, a significant decline in cell viability appeared for high flow rates and heterogeneous delivery, cargo- and cell-size dependent, was observed.

To enlarge cytosolic delivery by mechanoporation to a broad range of cargo molecules, Liu *et al.* recently presented a novel method with molecular size-independent delivery efficiency, showing the importance of timescale deformation and cell mechanical properties in mechanoporation.^23,24^ The molecules were loaded into cells by a phenomenon called cell volume exchange for convective transfer (cell VECT) enabled by rapidly passing cells through a microchannel, in which they undergo sudden deformations under several ridges. Cell VECT allowed delivery of different cargos with high efficiency regardless of molecule size and without impairing cell viability mostly in compliant cells. Notably, even though molecular loading was independent of molecules' size, it remained dependent on cell size and velocity with delivery efficiency varying with number of ridges and gap size. Thus, VECT is dependent on timescale of deformation (compression time) and cell mechanical properties,^23,24^ suggesting that the efficiency of the method might vary among different cells. Following the principle of volume exchange during fast deformation, Kizer *et al.* introduced a new method called hydroporator, based on the rapid hydrodynamic cell shearing inside a microfluidic device under high Reynolds number (Re > 10^2^, ESI,† relevant parameters). Increasing the flow rate increased delivery efficiency, but at the expense of decreased cell viability. Furthermore, the delivery efficiency was dependent on cell deformability and cargo size, and the delivery of different cargos was shown mostly on compliant cells.^12^ Mechanoporation methods have already shown their potentiality in biological and therapeutical applications such as the delivery of transcription factors,^10^ nanoparticles as drug nanocarriers,^25^ impermeable inhibitors,^18^ protein antigens,^19^ and Cas9–protein gRNA complexes.^16^ However, further technology development and investigation of cell physical properties are required to have a method that is equally applicable to the delivery of cargos in cells irrespective of their physical properties.

Towards this goal, recently, different constriction geometries have been developed.^25–27^ Lately, for such delivery platforms, the importance of cell elasticity has also been shown^27^ and more investigation towards parameters affecting the cell deformation is required. Furthermore, the potential application of these methods from basic biology to clinical applications, rely on the use of simple operation, low cost, flexible and reliable microfluidic platforms able to provide high delivery efficiency in high throughput manner independent of the cargo and cell types, while not significantly affecting the cell functionality.

We hypothesize that not only the extent of compression experienced by cells while flowing in a narrow constriction, but also cell mechanical properties combined with the cell status before squeezing (pre-deformed) as well as timescale deformation play an important role in the temporary disruption of the cell membrane. For a fixed flow condition and chip geometry, more compliant cells can more easily deform and move through a narrow constriction compared to stiff cells.^28,29^ Furthermore, recently, it has been shown that cell deformability/elasticity affects cell velocity in laminar flow.^30,31^ Thus, cells with different elasticity can flow differently in microchannels affecting cell membrane poration and the consequent intracellular delivery. Cell pre-deformation before the contact-based compression can be used to avoid mechanical shock on cells that approach narrow constrictions and to facilitate their transition reducing the impact on cell viability. This can enlarge the range of applicable pressure/flow and compression not affecting cell viability with a consequent improvement in delivery efficiency among cells with different physical properties. Pre-deformed cells can cross the microchannels length in shorter time, leading to shorter timescale deformation. Furthermore, pre-deforming cells with different elasticity can homogenize the way cells travel in microchannels and provide comparable treatment among cells with different mechanical properties.

Here, our method aims to provide a comparable and higher delivery efficiency among cells with different mechanical properties. We named the method progressive cell membrane mechanoporation (PM) and we developed it on a robust, low-cost and user-friendly PDMS-based microfluidic platform to spread its usage in different labs. Cells flowing in our PM device experience a multistage deformation. In particular, the cells are pre-deformed before they get squeezed in the narrow constrictions. A multistage cell deformation is achieved during cell flow by the combination of hydrodynamic forces in a microchannel with a width comparable to cell size, followed by a rapid contact-based compression in consecutive microchannels with a width smaller than the cell diameter. PM devices are produced by a standard single-layer PDMS process.^32^ The liquid flow inside the device is activated and controlled by a pressure-based microfluidic controller equipped with a flow sensor. Cells are passed through 60 parallel microchannels, each characterized by a gradual decrease in channel size to provide a gradual and progressive increase in cell strain. In each channel, cells are pre-deformed by hydrodynamic forces followed by contact-based compression. We show that the device supports high pressure (up to 5 bar) and flow rates (up to 1170 μL min^−1^). It allows processing a small volume of cell suspension (about 20 μL) with high-throughput (>10 000 cells per s) while cells maintain their viability and proliferation capacity. Furthermore, we take advantage of PM to investigate the influence of cell elasticity in the uptake of cargos with different molecular weights using two cell types with the same size but a different Young's modulus in devices with different channel geometries. We confirm that membrane poration activated by mechanical forces is enhanced under fast deformation (high cell velocity). Compared to the shear-induced and contact-mediated squeezing methods, PM provides high delivery efficiency among cells with different mechanical properties, without affecting their viability and proliferation rates. We show that the decrease in delivery efficiency for cargos of increasing size is affected by cell mechanical properties, where the loading of bigger molecules is favourable in stiffer cells, opposite to the behaviour observed for small molecules. Importantly, PM enables the functional delivery of large (190 kDa) gene-editing competent Cas9 protein–single guide RNA ribonucleoprotein complexes (Cas9–sgRNA RNPs) crucial for upcoming biomedical and clinical applications.

The rational design of our system and the reliable operation and easy-assembly of the PDMS-based chip is optimized to achieve multistage cell deformation through combination of features that provide an improved and unique intracellular delivery by PM. This represents a further step towards the goal of improving mechanoporation performance irrespective of cell physical properties and to widespread the use of the method.

## Results

### Device operation by a pressure and flow microfluidic controller

It is important to provide a microfluidic platform to allow the operation of our chip under a broad range of pressure/flow conditions in a reliable, reproducible and easy manner apt to support on-chip cell membrane mechanoporation.

**Fig. 1:**
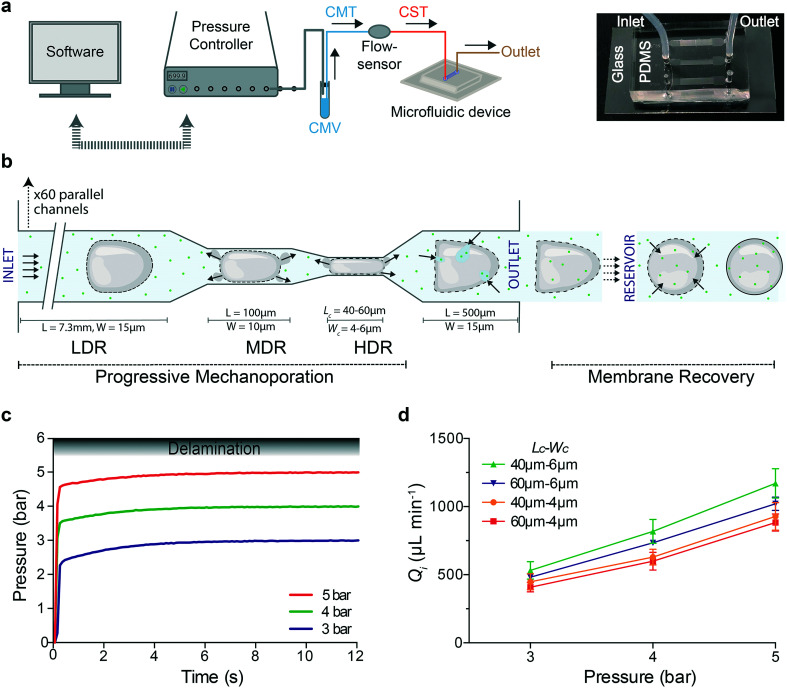
Progressive mechanoporation: rapid and gradual cell deformation under controlled pressure and flow rate a) Left: schematic representation of the PDMS-based microfluidic platform for progressive mechanoporation (PM). The set-up consists of a pressure controller connected to a vial containing CO_2_-independent cell culture media (CMV) which is further connected to a flow sensor *via* a tubing filled with the same media (CMT). Another tubing filled with cell suspension (CST) is attached between the flow sensor and microfluidic device inlet. To collect the mechanoporated cells, a tubing is connected at the outlet of the device. Right: Picture of the microfluidic chip made of a micro structured PDMS element bonded on a glass coverslip. Each chip includes three microfluidic devices. FEP tubing are inserted in correspondence of the inlet and outlet chambers. b) Schematic representation of the progressive cell membrane mechanoporation during cell flow inside a single channel and cell membrane recovery in the collection tube (reservoir). The channel comprises of three deformation regions: low (LDR), medium (MDR) and high deformation region (HDR). For each region, length (*L*) and width (*W*) of the corresponding channel are indicated. The arrows in the MDR, HDR and before the outlet, indicate the cell volume exchange resulting in convective molecular transport into cell cytoplasm at the outlet. The arrows in the reservoir show the diffusion of molecules from the surrounding medium to the cell cytosol before cell membrane repair. c) Pressure stabilization over time for four different operating pressures (3, 4 and 5 bar). The gradient grey zone shows the device delamination for operating pressure higher than 5.5 bar. d) Flow rate at the device inlet, *Q*_i_, as function of the applied pressure for four different device geometries (*L*_c_–*W*_c_ = 40–6μm; 60–6μm; 40–4μm; 60–4μm). The mean values and SD of three independent experiments are plotted.

We assembled a microfluidic platform for high-throughput processing of small sample volumes under stable conditions. To ensure that the flow rate is not varying while the cells are processed, we used a pressure-based microfluidic controller (Fluigent MFCS™ EX) interfaced with a flow sensor and a commercial software (MAESFLO™) to control pressure and measure flow rate in real time (Fig. 1a). A vial containing the same media (CMV) was connected to the pressure controller and to a flow sensor *via* a tubing filled with the same media (CMT). To minimize the sample volume (about 20 μL), the cell suspension was loaded in the tubing (CST) connected between the flow sensor and the device inlet. Subsequently, processed cells were collected through a tubing at the outlet of the device (Fig. 1a). In each device, the cell suspension flows from inlet towards outlet moving through 60 parallel channels (Fig. S1†), where each channel is characterized by three regions that are able to act on cell deformation with different strength (Fig. 1b): 1) low deformation region (LDR): 15 μm wide, 7.3 mm long, 2) medium deformation region (MDR): 10 μm wide, 100 μm long, 3) high deformation region (HDR): 4 or 6 μm wide (*W*_c_), 40 or 60 μm long (*L*_c_), which is 550 μm far from the outlet chamber. The LDR allows cell pre-deformation before their compression in the HDR. Microfluidic devices are denoted by *L*_c_–*W*_c_ based on their HDR dimensions. The flow inside the device is activated by changing the pressure inside the vial (CMV) and the corresponding flow rate is recorded.

Here, the devices are produced by simple air plasma activated bonding of a micro structured PDMS element on a flat glass coverslip able to support high applied pressure without delamination. Optimal combination of punched holes (1 mm) in correspondence of the inlet chamber and tubing size (CST with O.D. 1.5 mm) guarantees a watertight connection. The same results were obtained before by using a second PDMS casting, making the fabrication process more complex and time consuming.^21^ A combination of 60 parallel channels with a pressure-based microfluidic controller ensures that even if one or a few channels are clogged at the narrow constrictions, the pressure and eventually the exerted forces in an individual unclogged channel will remain the same. Furthermore, the compact geometry of our PDMS-based device allows the integration of three devices in one chip (Fig. 1a). The chip is easy to fabricate and cheap and it can be replaced with a new one after each process, avoiding cross-contamination among different experiments.

Our device is able to support pressure up to 5 bar (flow rate up to 1170 μL min^−1^). Beyond 5.5 bar, delamination between the PDMS element and glass was observed (Fig. 1c). The flow rate was monitored in real time during the sample processing for three different applied pressures (3, 4 and 5 bar) and four different devices (Fig. 1d). The Reynolds number (Re), the fluid shear stress (*τ*) and the mean cell velocity (ESI†) for different devices and applied pressures were ranging from 6.9–19.6, 149.5–428.5 N m^−2^ and 391–1047 mm s^−1^, respectively (Table S1†). The time for a cell to cross the entire length of the channel (8 mm) was 8–18 ms and the transition time in the MDR–HDR for an operating pressure of 3 bar was lower than 0.7 ms.

We demonstrate that our platform supports high pressure and is able to provide high flow rates with fast stabilization time, fundamental for processing small sample volume in a reproducible and reliable manner. Cells moving through the channel length experience a multistage deformation (Fig. S1 and ESI† Video) depending on the cell diameter/channel width ratio, cell mechanical properties and cell velocity. In particular, the cells are pre-deformed in the LDR (channel width comparable to the cell diameter) before their contact-based compression in the narrow constrictions (channels width smaller than the cell diameter). Membrane pores can already be formed in the LDR mostly due to hydrodynamic forces. Exactly, cells flowing in the LDR are subjected to high shear forces^14^ (up to 428.5 N m^−2^) and they are deformed under a flow regime dominated by inertial forces^12^ (Re > 1). In the MDR–HDR cells undergo high mechanical compression that can enlarge and enrich pores. At the same time volume exchange under fast deformation^23,24^ (<0.7 ms) can be favoured through the opened pores (Fig. 1b). The overall molecular uptake is the result of convective and diffusive transport of molecules from the cell surrounding to its interior through pores opened by hydrodynamic forces and contact-mediated cell compression. The resulting strain on the cells will be dependent not only on their size, but also on their mechanical properties, affecting both cell deformation^33^ and membrane poration^27^ and repair.^34,35^

### Analysis of cell size and elasticity

**Fig. 2:**
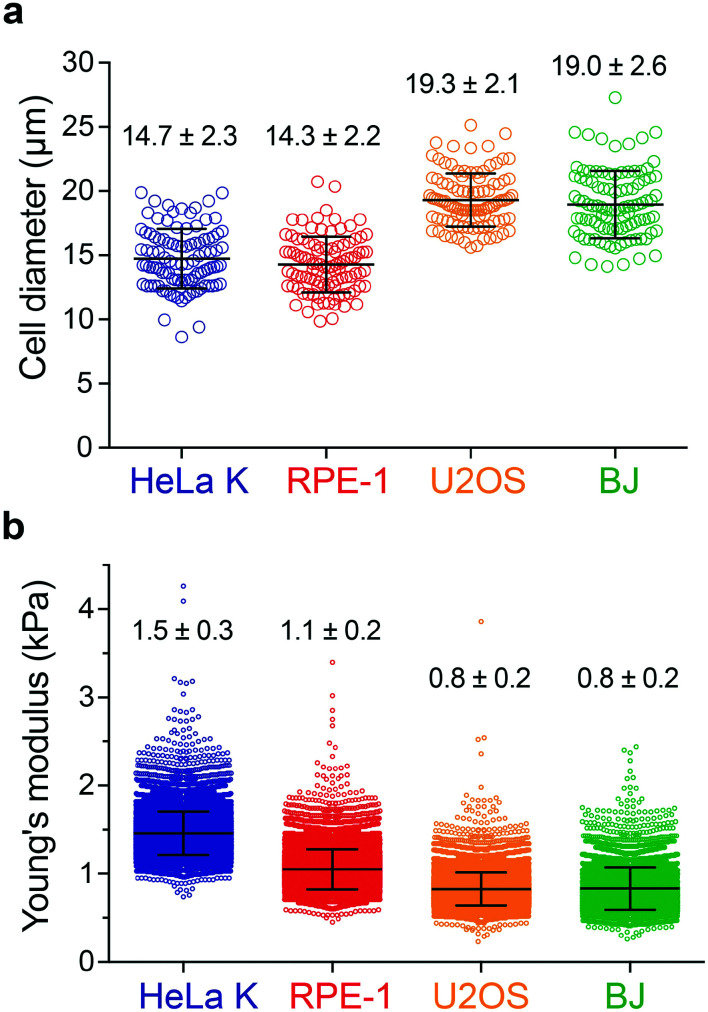
Analysis of cell size and elasticity. a) Cell diameter distribution for four different cell types: HeLa K, RPE-1, U2OS and BJ. b) Young's modulus distribution of HeLa K, RPE-1, U2OS and BJ cells measured by real-time deformability cytometry (RT-DC). The mean and SD are plotted.

To assess the impact of cell mechanics on the extent of permeabilization, two different cell types were selected, HeLa cells (Kyoto strain, HeLa K) and retina pigment epithelial cells (hTERT RPE-1). In addition, BJ fibroblasts and U2OS cells were included, respectively, for a direct comparison with a previous study^21^ and for characterizing a cellular model employed here to assess the applicability of PM in cell-based therapies (see Fig. 6). The size distribution of rounded cells was measured by bright-field image analysis determining their diameter after trypsin-mediated detachment (Fig. 2a). Cell elasticity was measured using real-time deformability cytometry (RT-DC) (Fig. 2b and S2†), which relies on flowing cells through a microchannel where the hydrodynamic shear-induced deformation of a cell is analysed at throughput of up to 1000 cells per s in real-time.^36^ Together, this revealed that RPE-1 and HeLa K cells have a similar diameter of (14.3 ± 2.2 μm and 14.7 ± 2.3 μm) but differ in elasticity (Young's modulus of 1.1 ± 0.2 kPa *versus* 1.5 ± 0.3 kPa, respectively). In contrast, the larger BJ fibroblasts and U2OS cells have similar size (19.0 ± 2.6 μm and 19.3 ± 2.1 μm) and elasticity (Young's modulus of 0.8 ± 0.2 kPa for both) (Fig. 2).

Delivery efficiency can vary with cell size, where larger cells can deform more in the three deformation regions, when travelling along the channel. In addition to the cell size, depending on their elasticity, cells deform differently (Fig. S2†) which can lead to differences in the delivery efficiency. Thus, analysing RPE-1 and HeLa K (comparable size, different elasticity) allows to decouple these two parameters providing insights on the impact of cell elasticity on delivery efficiency.

### Intracellular delivery of small molecules into cells with comparable size and different elasticity

Biological studies and clinical applications often require the intracellular delivery of small impermeable cargo molecules such as intracellular probes, dyes, inhibitors, or peptides, with molecular weight <5 kDa.^8^ Therefore, we first assessed how efficiently PM devices deliver small molecules, specifically 4 kDa FITC-dextran, mimicking the size of above-mentioned molecules into different cell types.

**Fig. 3:**
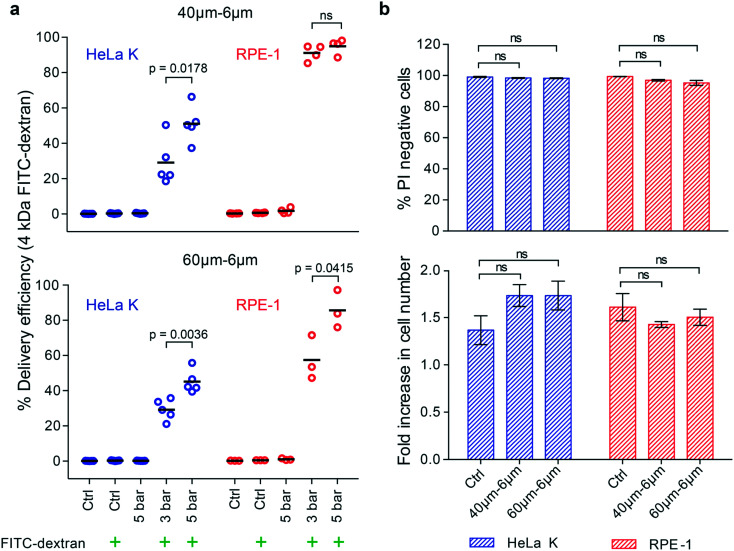
Intracellular delivery of small molecules into cells with comparable size and different elasticity. a) Delivery efficiency of 4 kDa FITC-dextran in HeLa K and RPE-1 cells, obtained by using 40–6μm (*L*_c_–*W*_c_) (top) and 60–6μm (*L*_c_–*W*_c_) (bottom) devices. The delivery efficiency was measured by FACS for the following conditions: not treated cells (Ctrl); cells treated with operating pressure of 3 bar and 5 bar. The symbol (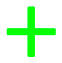) represents the addition of 4 kDa FITC-dextran to the cell suspension. Individual measurements (circles) and mean values (line) are reported. Significance between 3 bar and 5 bar samples according to an unpaired *T*-test (ns = not significant). b) Cell viability of HeLa K and RPE-1 cells represented as propidium iodide (PI) negative cells (top), measured by FACS analysis of cells stained with PI directly after PM in 40–6μm and 60–6μm (*L*_c_–*W*_c_) devices (pressure 5 bar) and without PM (Ctrl). Fold increase in cell number (bottom) comparing HeLa K and RPE-1 cells 24 hours after PM in 40–6μm and 60–6μm (*L*_c_–*W*_c_) devices and without PM (Ctrl) measured by bright-field microscopy. The mean values and SD of three independent experiments are plotted. Significance according to Kruskal–Wallis one-way analysis of variance (ns = not significant).

The delivery efficiency of such molecules (4 kDa FITC-dextran) into HeLa K and RPE-1 cells was measured by fluorescence activated cell sorting (FACS) analysis. Two different devices, with same *W*_c_ of 6 μm and different *L*_c_ of 40 μm and 60 μm were used under two pressure conditions, 3 bar and 5 bar. The ratio between the cell diameter and constriction width was calculated and it ranges from 1.4 to 3.5 for both cell types. To gate for PM-specific uptake of FITC-dextran, cells not passed through the device (Ctrl) or exposed to 4 kDa FITC-dextran without PM (Ctrl + FITC-dextran) were analysed in parallel (Fig. S3†). Delivery efficiency was calculated as the ratio between number of 4 kDa FITC-dextran positive cells and total number of analysed cells. While 40–6μm and 60–6μm (*L*_c_–*W*_c_) devices enabled the delivery of 4 kDa FITC-dextran into both, HeLa K and RPE-1 cells (Fig. 3a), differences in delivery efficiency were observed as a function of the operating pressure and cell type. Increasing the pressure (from 3 to 5 bar) increased the number of FITC-positive HeLa K cells in both devices 40–6μm (*L*_c_–*W*_c_) (from 29% to 51%) and 60–6μm (*L*_c_–*W*_c_) (from 29% to 45%), in agreement with a previously shown relation between pressure and delivery efficiency.^10^ A similar trend was observed with RPE-1 cells in the 60–6μm (*L*_c_–*W*_c_) device (from 57% to 86%). In contrast, with the previously reported relation between *L*_c_ and delivery efficiency, we observed for the RPE-1 cells a higher delivery efficiency in the shorter device (up to 95%) compared to the longer one (from 57% to 86%) (Fig. 3a and S4†). Besides, variability in delivery efficiency among different measurements was observed (Fig. 3) which could be due to heterogeneity within the cell population (Fig. 2). Notably, for both the devices delivery efficiency was higher into RPE-1 that are more compliant than HeLa K cells, even though the diameter of both cell types was almost identical (Fig. 2a).

We also assessed how PM affects cell viability by staining for dead cells with propidium iodide (PI) directly after the treatment with device using untreated cells as a negative control (Ctrl). Even at the highest applied pressure (5 bar), we observed almost no dead cells directly after treatment, neither in HeLa K nor in RPE-1 cells (Fig. 3b). Importantly, the proliferation rate of cells during 24 hours after the treatment was comparable to untreated control cells (Fig. 3b) suggesting that the mechanical stresses experienced by cells during PM had no longer term adverse effects on overall cell homeostasis.

We conclude that PM with 6 μm wide constrictions can deliver small molecules with up to 95% efficiency into RPE-1 cells independent of the applied pressure and without affecting cell viability and proliferation. For HeLa K cells, comparable in size but stiffer than RPE-1, we found a lower delivery efficiency increasing with the increase of the applied pressure. This highlights cell elasticity as an intrinsic physical property important to consider for efficient mechanoporation.

### Progressive mechanoporation in narrower constriction enhances delivery efficiency of small molecules into stiffer cells

**Fig. 4:**
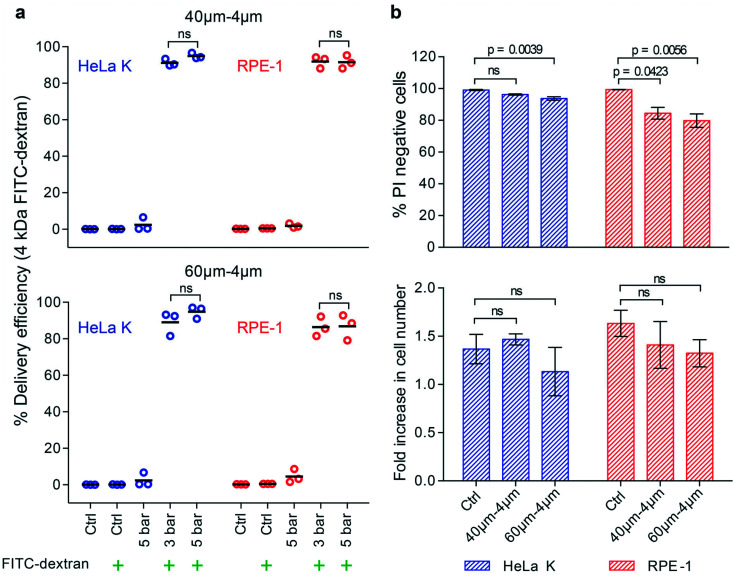
Progressive mechanoporation in narrower constriction enhances delivery efficiency of small molecules into stiffer cells. a) Delivery efficiency of 4 kDa FITC-dextran in HeLa K and RPE-1 cells, obtained by using 40–4μm (*L*_c_–*W*_c_) (top) and 60–4μm (*L*_c_–*W*_c_) (bottom) devices. The delivery efficiency was measured by FACS for the following conditions: not treated cells (Ctrl); cells treated with operating pressure of 3 bar and 5 bar. The symbol (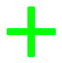) represents the addition of 4 kDa FITC-dextran to the cell suspension. Individual measurements (circles) and mean values (line) are reported. Significance between 3 bar and 5 bar samples according to an unpaired *T*-test (ns = not significant). b) Cell viability of HeLa K and RPE-1 cells represented as propidium iodide (PI) negative cells (top), measured by FACS analysis of cells stained with PI directly after PM in 40–4μm and 60–4μm (*L*_c_–*W*_c_) devices (pressure 3 bar) and without PM (Ctrl). Fold increase in cell number (bottom) comparing HeLa K and RPE-1 cells 24 hours after PM in 40–4μm and 60–4μm (*L*_c_–*W*_c_) devices and without PM (Ctrl) measured by bright-field microscopy. The mean values and SD of three independent experiments are plotted and *p* values are indicated. Significance according to Kruskal–Wallis one-way analysis of variance (ns = not significant).

We observed that at the same ratio of cell diameter to constriction width delivery was more efficient into softer RPE-1 than into stiffer HeLa K cells (Fig. 3a). To increase the intracellular delivery into stiffer cells the contribution of progressive mechanoporation was enhanced by decreasing *W*_c_ to 4 μm. The ratio between the cell diameter and the reduced constriction width was calculated and it ranges from 2.2 to 5.2 for both cell types. We observed that 40–4μm (*L*_c_–*W*_c_) device was able to provide higher delivery efficiency for HeLa K (91–95%) comparable to the one of RPE-1 (both 92%) for both the applied pressure (3–5 bar), (Fig. 4a). This demonstrates that adapting device geometry is a strategy to efficiently deliver small cargos at lower pressure independent of the cell elasticity. For the longer constriction (60–4μm (*L*_c_–*W*_c_)), we measured a slightly lower delivery efficiency, from 89–95% for HeLa K and 86–87% for RPE-1 cells (Fig. 4a and S4†). A pressure of 3 bar was sufficient to reach high intracellular delivery providing high viability for HeLa K (>90%) as well as for RPE-1 (>80%) cells and did not significantly affect proliferation capacity during the subsequent 24 hours (Fig. 4b). The longer device (60–4μm (*L*_c_–*W*_c_)) decreased slightly cell viability directly after PM as reported previously.^10^ Thus, the pressure of 3 bar combined with 4 μm *W*_c_ provided a perfect compromise between delivery efficiency, cell viability and proliferation capacity.

### Intracellular delivery of bigger molecules and biologically relevant cargo

**Fig. 5:**
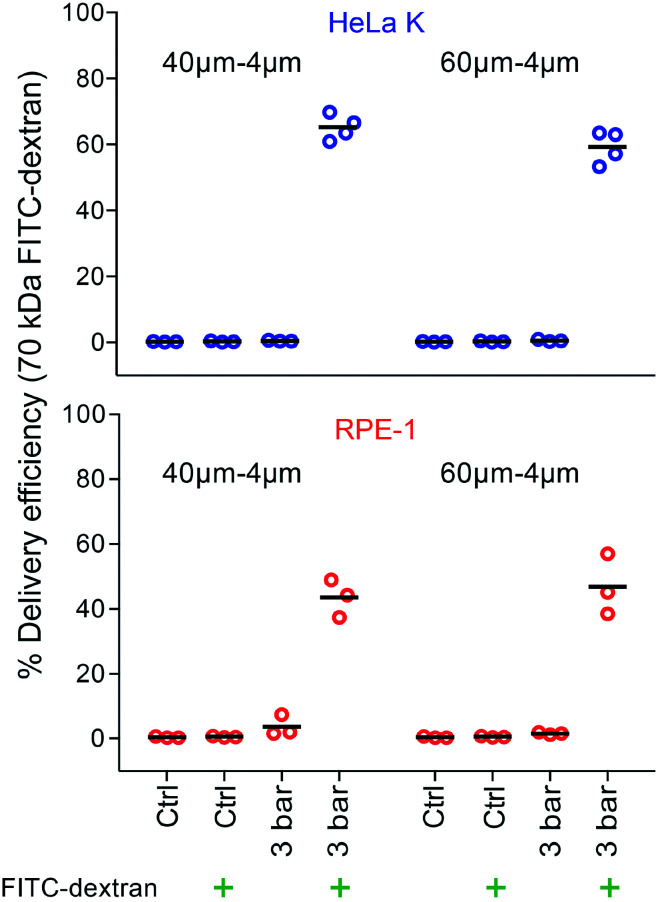
Intracellular delivery of bigger molecules in a size range of biologically relevant cargo. Delivery efficiency of 70 kDa FITC-dextran in HeLa K (top) and RPE-1 (bottom) cells, obtained by using 40–4μm and 60–4μm (*L*_c_–*W*_c_) devices. The delivery efficiency was measured by FACS for the following conditions: not treated cells (Ctrl); cells treated with operating pressure of 3 bar. The symbol (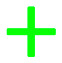) represents the addition of 70 kDa FITC-dextran to the cell suspension. Individual measurements (circles) and mean values (line) are reported.

Applying an optimal constriction width (4 μm) and pressure (3 bar), we assessed the ability of PM to deliver larger cargo molecules that were similar to the size of therapeutically-relevant molecules, such as antibodies, transcription factors or CRISPR/Cas9 gene-editing complexes. Firstly, the delivery of 70 kDa FITC-dextran was tested. We reached delivery efficiency of (65%, 59%) for HeLa K and (44%, 47%) for RPE-1 cells (Fig. 5) by using device with different *L*_c_ (40 μm, 60 μm). This demonstrates the ability of PM to deliver larger molecules, albeit with reduced capability compared to the smaller ones.

**Fig. 6:**
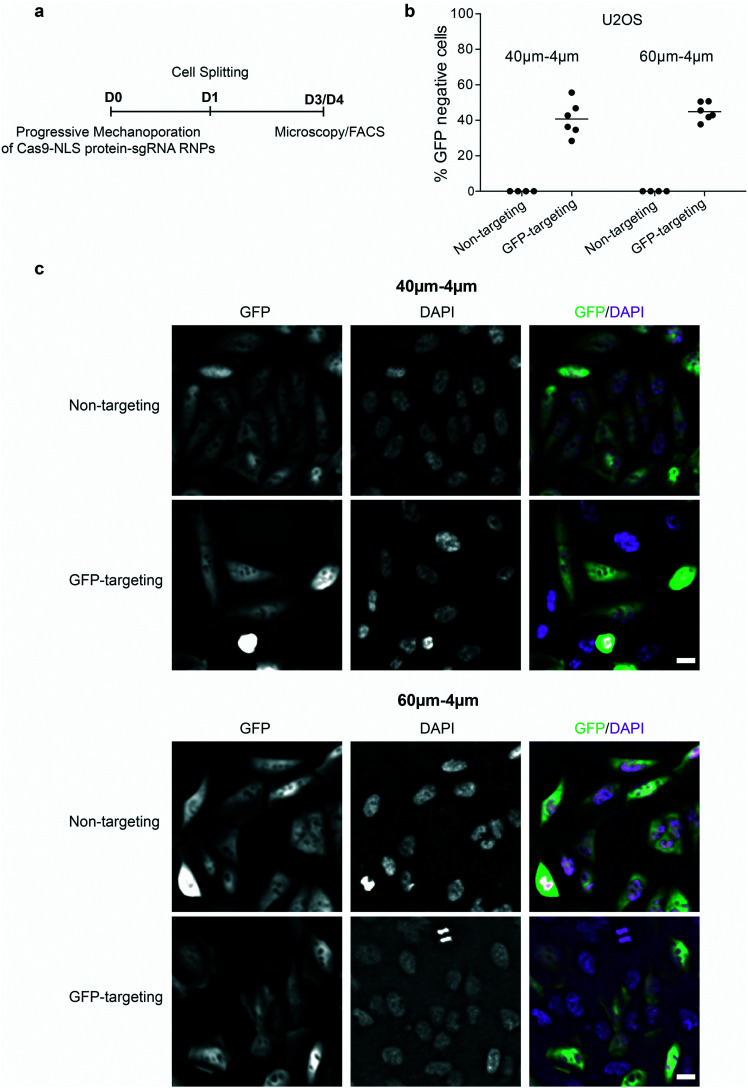
Successful delivery of Cas9–sgRNA RNPs. a) Scheme of the Cas9–NLS protein-sgRNA RNPs delivery into U2OS cells (D0 = day 0), cell splitting (D1 = day 1), the microscopy (D3 = day 3) and FACS analysis (D4 = day 4). b) Delivery efficiency of Cas9–NLS protein–sgRNA RNPs targeting GFP (GFP targeting) or non-targeting in U2OS, obtained by using 40–4μm and 60–4μm (*L*_c_–*W*_c_) devices with operating pressure of 3 bar and analysed by FACS as percentage of GFP negative cells. Individual measurements (circles) and mean values (line) are reported. The data were obtained from two independent experiments (with three technical repeats for Cas9–sgRNA RNPs targeting GFP and two technical repeats for Cas9–sgRNA RNPs non-targeting within one independent experiment). c) Fluorescent images of cells after delivery of non-targeting or GFP-targeting Cas9–sgRNA RNPs as indicated in (a), by using 40–4μm (*L*_c_–*W*_c_) (top) and 60–4μm (*L*_c_–*W*_c_) (bottom) devices. DAPI was used for DNA staining. The scale bar is 20 μm.

To demonstrate the potential of PM for upcoming cell therapies based on gene-editing, we aimed to deliver a functional 190 kDa large Cas9 protein–single guide RNA ribonucleoprotein complex (Cas9–sgRNA RNP) into cells. Therefore, we used PM to deliver recombinant nuclear-localized Cas9 protein (Cas9–NLS) complexed with a sgRNA targeting the green fluorescent protein (GFP) into an U2OS reporter cell line containing single copy of the GFP gene.^37^ As a control, non-targeting sgRNA in complex with Cas9–NLS was employed. The percentage of GFP negative cells was analysed by FACS and fluorescence microscopy (Fig. 6a). Loss of GFP fluorescence is indicative of functional Cas9–sgRNA RNPs targeting GFP delivery and subsequent knock out of the GFP gene. Indeed, delivery of Cas9–sgRNA RNPs targeting GFP but not a non-targeting control resulted in 41% and 45% GFP negative cells using 40–4μm and 60–4μm (*L*_c_–*W*_c_) devices, respectively (Fig. 6b and c). Notably, cell mechanoporation itself did not induce any changes in GFP levels (Fig. S5†).

Thus, our PDMS-based progressive mechanoporation platform enables efficient and functional delivery of cargo molecules with high molecular weights highlighting its applicability for CRISPR/Cas9-mediated gene-editing.

## Discussion

Mechanoporation is a powerful approach that has been realized in different ways to permeate cell membrane for intracellular molecular loading. Methods based on shear-induced or contact-mediated cell deformation, employing different materials, device geometry and flow or pressure controller, have been developed in recent years to widen the applicability to different cell and cargo types.^1,8–21,23–27,38^ In previous mechanoporation methods, the flow inside the microfluidic chip has been controlled by either pressure-driven^10^ (constant pressure) or a syringe-driven^21^ (constant flow) system. Pressure-driven flow provides faster response and stabilization.^22^ However, for a constant applied pressure flow rate might change during the process and a measurement/control of the flow rate is needed to ensure process stability over the experimental time. Furthermore, pneumatic pressure controllers have been mostly used in combination with silicon-based chips,^10,11^ able to support high applied pressure, but the time-consuming and high cost of the chip production, prerogative of specialized facilities, limit their usage.^39,40^ The extension of pressure controller to PDMS-based chip would be ideal to widespread the use of the method, but not trivial. It requires watertight connection and good bonding strength to support the operation of the chip under high pressure/flow in applications such as cell mechanoporation.

In this study, we have presented a novel method named progressive mechanoporation (PM) implemented on a PDMS-based microfluidic platform for intracellular delivery. It combines shear-induced and contact-mediated membrane poration *via* multistage cell deformation in a PDMS-based device controlled through a pressure and flow microfluidic controller for a reliable and reproducible cell treatment (Fig. 1a). In contrast with similar devices in the literature, employing channels with only one high shear or compression region,^10,21^ we designed a device combining cell shearing dominated by inertial forces (LDR) with two consecutive short compression regions (MDR–HDR) inducing a progressive increase in cell deformation (Fig. 1b). The pre-deformation of cells in the LDR combined with the gradual channel width variation compared to the abrupt change previously used,^10,21^ can avoid mechanical shock on cells, enhancing their viability. Moreover, it can improve the performance of the method across different cell types through molecular loading aided by the combination of convective and diffusive transport through membrane pores opened and enriched along the microchannels (Fig. 1b).

The devices are produced by bonding a micro-structured PDMS element on a glass coverslip, where the proper combination of punched holes and tubing size allows to obtain a watertight and leakproof device. Lam *et al.*^21^ used a second PDMS casting to obtain a chip that can support a maximum flow rate of 750 μL min^−1^. In this case the flow inside the chip was controlled by a syringe pump which led to fluctuations and longer stabilization time.^21,22^ Even if our device has a higher flow resistance, due to the smaller size of the microchannels, it can support pressure and flow rate, respectively up to 5 bar; 1170 μL min^−1^. Furthermore, the device operation by a microfluidic controller, equipped with a flow sensor allows to process low sample volume (about 20 μL) with high-throughput (>10 000 cells per s), under high and stable pressure/flow condition. This results in a more homogeneous cell treatment across the parallel channels, enhancing the reproducibility of the method.

Similar to our approach, Lam *et al.* employed a PDMS device with parallel channels, but using a single contact-mediated squeezing region. They reported the 6 μm wide and 30 μm long constriction geometry to be optimal to achieve high delivery efficiency into BJ fibroblasts.^21^ Referring to this, we designed our devices with the HDR, characterized by a similar constriction width (*W*_c_ = 6 μm), but greater length (*L*_c_ = 40 μm or 60 μm), expecting a higher delivery efficiency for the longer constriction, as previously reported.^10^ We selected two cell types, HeLa K and RPE-1 cells, with a comparable diameter but different Young's modulus to investigate the impact of cell elasticity on intracellular delivery. For comparison with results reported by Lam *et al.*, the size and Young's modulus of BJ fibroblasts were also determined (Fig. 2).

In agreement with previous studies, for a fixed device geometry, the delivery efficiency of 4 kDa FITC-dextran into both cell types increased with the increase of the applied pressure. However, contrary to previous contact-mediated intracellular delivery methods,^10^ the delivery efficiency increased when reducing the constriction length from 60 to 40 μm (Fig. 3a and S4†). This different behaviour can be explained considering that our PM method combines both, shear-induced and contact-mediated membrane poration. Given the Hagen–Poiseuille equation, the flow resistance is directly proportional to the channel length,^41^ hence for a fixed pressure, the flow rate is higher in shorter constriction. Higher flow rate (from 400 to 1170 μL min^−1^) provided higher shear stress (from 149.5–428.5 N m^−2^) and Reynolds number (from 6.9 to 19.6) (Table S1†) in the LDR, leading to cell pre-deformation and cell membrane poration, in agreement with previous reports employing high shear stress (>200 N m^−2^) in microchannels^14^ and rapid hydrodynamic cell deformation under high Reynolds number (Re = 189).^12^ Moreover, the opened pores facilitate cytosol exchange during cell squeezing in MDR–HDR, where VECT is enhanced by fast deformation,^23,24^ in our case promoted by higher cell velocity and shorter constriction (Fig. 1b). While Lam *et al.* observed for BJ fibroblasts a significant decline in cell viability at 750 μL min^−1^, our results show a viability of higher than 95% and a good proliferation rate under high pressure (5 bar) and flow rate (1021–1170 μL min^−1^), (Fig. 3b).

Remarkably, we observed a dependence of delivery efficiency on cell elasticity. While the shorter constriction already enabled higher delivery of small 4 kDa FITC-dextran into RPE-1 cells, the molecular loading into stiffer HeLa K cells was less efficient and dependent on the operating pressure (Fig. 3a). This result confirms previous observations, where delivery efficiency of small cargo dropped to 25–35% into less deformable stiffer cells.^12^ In agreement with our results, a recent study has also reported more internalization of small molecules by inhibiting the actin polymerization decreasing the elasticity of cells.^27^ Therefore, the success in membrane poration for a fixed stress is dependent on the duration and extent of induced cell deformation, which can vary among different cell types and within the same cell population due to intrinsic cell to cell heterogeneity not only in size but also in elasticity (Fig. 2).

To enhance the delivery efficiency into stiffer cells by increasing their deformation the constriction in the HDR was reduced to ∼1/3 of the cell diameter (4 μm). Previous works have shown that cell volume change under contact-mediated cell compression increased under higher cell strain^23,24^ promoting convective transport. In the same fashion, higher cell strain in smaller constrictions enhanced membrane poration as reported in previous studies facilitating subsequent diffusive intracellular molecular loading.^10,11^ The use of devices with smaller constriction drastically increased the delivery efficiency of small cargo into both cell types with values higher than 90% independent of the constriction length and operating pressure (or flow rate, or cell velocity) (Fig. 4a and S4†). We observed an increased variability in delivery efficiency among different measurements for the chips with a constriction width of 6 μm compared to 4 μm constriction chips (compare Fig. 3 to 4). The difference might be due to size and elasticity heterogeneity within the cell population (Fig. 2). It suggests that a certain threshold of the exerted forces and time they are applied has to be overcome to have comparable cell membrane poration among cells with different physical properties. This threshold is lower for more compliant cells showing a higher uptake of the molecules (>90%) already within the 40–6μm (*L*_c_–*W*_c_) device (Fig. 3a).

Compared to the device geometry employed by Lam *et al.*^21^ and Sharei *et al.*^10^ our progressive mechanoporation, provides a higher delivery efficiency (70–75% *versus* >90% in our study), already under operating pressure of 3 bar (flow rate 448 μL min^−1^, cell velocity 514 mm s^−1^) (Table S1†) without significantly reducing viability (>80%) and proliferation. Kizer *et al.* reported for 3–5 kDa dextran in compliant K562 cells (Young's modulus = 0.4 kPa^23^) a delivery efficiency of about 90% with a viability of *circa* 80% for high value of Reynolds number (Re = 189). However, the delivery efficiency was drastically reduced to 25–35% for less deformable stiffer cells (*i.e.* HeLa, MCF7).^12,42^ Our method provides higher delivery efficiency among cells with different elasticity under lower Reynolds number (Re = 6.9–15.6) (Fig. 4a, Table S1†). Comparable results have been reported by Lam *et al.* with BJ fibroblasts that are being bigger and softer than HeLa K or RPE-1 cells and thus can be easily porated.^21^ Sharei *et al.* showed an efficiency delivery lower than 80% on HeLa K cells for a 40–6μm (*L*_c_–*W*_c_) device geometry.^10^ Our results demonstrate that a strategically designed device geometry provides a comparatively high delivery efficiency of small impermeable molecules into different cell types despite their differences in mechanical properties while keeping a high value of cell viability and proliferation (Fig. 4b). We show that the same device geometry (*W*_c_ = 4 μm) can be successfully used for the intracellular delivery of comparatively bigger molecules (70 kDa FITC-dextran), but with lower efficiency compared to small molecules (Fig. 5). This has been mostly attributed to variation of diffusive transport dependent on cargo size,^10,15,21,27^ where small molecule diffuses more rapidly into the cell cytosol than larger molecules. Nevertheless, the delivery efficiency of 70 kDa FITC-dextran into HeLa K cells was 20% higher than the one reported by Sharei *et al.* and Lam *et al.* using on HeLa cells^10^ and BJ fibroblasts,^21^ respectively. Hence, we believe that the better performance cannot be ascribed only to intrinsic physical properties of cells but are mostly due to the enhanced cell permeability by the progressive mechanoporation.

Opposite to small molecules (Fig. 4) our results indicate that bigger cargos are more efficiently delivered into stiffer HeLa K than into more compliant RPE-1 cells (Fig. 5). A possible explanation is the different size, number and lifetime of opened pores as previously described.^10,21,26^ It has been shown that small pores can be resealed by membrane tension whereas larger pores (>0.2 μm) tend to be detected and repaired more quickly through calcium-activated sealing processes.^15,27,34,35^ A previous study suggested that pores created by cell squeezing with a size of more than 200 nm exhibit a reduced lifetime to 15–30 s in the presence of Ca^2+^ ions.^15^ During membrane disruptions of more than a few hundred nanometres, the existing membrane tension opposes the spontaneous pore resealing. Hence, calcium-activated exocytosis as well as remodelling of cytoskeleton accompanied by vesicular transport to the membrane governs the reduction of membrane tension while facilitating the pore resealing.^34,35^ In the cytoskeleton structure, mainly the amount of actin and its level of cross-linking contribute significantly to cell elasticity.^43^ We presume that opened pores in stiffer cells last longer due to a more densely packed cytoskeleton resulting in a longer time for actomyosin contraction and actin depolymerization necessary for pore resealing through vesicle–vesicle and vesicle–plasma membrane fusion.^43,44^ The prolonged pore opening grants more time for the larger molecules to diffuse into the cytoplasm, leading to higher delivery efficiency in stiffer cells (here, HeLa K).

Even though, plasmid DNA and nanoparticles are often much larger than proteins their more uniform chemical features greatly facilitate their delivery, *e.g.*, by lipofection across membranes. In contrast, the delivery of proteins or protein nucleotide complexes such as Cas9–sgRNA RNPs is much more challenging due to their highly varying chemical properties.^6,8,45,46^ Electroporation of proteins recently gained attraction; however, at least in human T cells electroporation causes the misexpression of 34% of all genes and unspecific upregulation of cytokines.^16^ Our finding that PM enables Cas9–sgRNA RNP-mediated gene editing in 45% of treated cells (Fig. 6) indicates the potential of PM to deliver large biologically active proteins or protein nucleotide complexes into cells for clinical applications. Since cells can repair the CRISPR/Cas9-induced double strand breaks without disrupting the targeted gene, the delivery efficiency of Cas9–sgRNA RNPs was likely even higher. Thus, our results are comparable to the delivery of Cas9–gRNA RNPs in human T cells where, 47% of gene-editing efficiency, targeting the PD1 gene, was achieved using silicon-based devices.^16^ Thus, PM can be used for gene-based therapy and our results show comparable delivery efficiency to that of complex and costly silicon-based devices.^16^

The rational design of our system in combination with the multistage cell deformation is a novel and unique approach to improve mechanoporation performance and to overcome differences due to variation in cell mechanical properties. Furthermore, the PDMS-based chip is optically accessible and further functionalities can be envisioned including real-time detection of cell pre-deformation. This can help to automatically adjust the pressure/flow to a certain value apt to provide specific cell pre-deformation to maximize the delivery efficiency and cell viability among different cell types in the future. Eventually, applications for the PM microfluidic platform can be envisioned, *i.e.*, to study cellular processes involved in mechanotransduction such as molecular turnover, cytoskeletal rearrangement, stem cell differentiation which are crucial exploration for regenerative therapies in the future.^47^

## Conclusion

Here, we present progressive mechanoporation (PM) as a novel and promising intracellular delivery method for processing small sample volume (about 20 μL) with high-throughput (>10 000 cells per s) in a reproducible and reliable manner. The gradual and fast (<0.7 ms) multistage deformation of cells activated by the combination of hydrodynamic forces and contact-mediated compression along rationally constricted microchannels provides high delivery efficiency among cells with different mechanical properties without adverse effects on cell viability and proliferation capacity. The easy, fast and low-cost production of the devices by single-layer PDMS ensures widespread applicability to academic labs. Furthermore, the easy operation of the platform makes it suitable for biomedical, biological and clinical applications. Due to its transparency, the presented device is suitable for real-time cell analysis by optical methods to enable future investigations into the relationship between cell deformation and performance on intracellular delivery. Finally, the ability of PM to efficiently deliver functional Cas9–sgRNA RNP complexes into cells opens up therapeutical opportunities for future CRISPR/Cas9 mediated gene-editing approaches, *e.g.* to easily access the cells of the hematopoietic system.

Although high delivery efficiency has been obtained for small cargo, bigger molecules showed a lower influx inside the cell cytosol, mostly diffusion-driven. In the future, the contribution of transport due to cell volume change could be enhanced by including more constrictions in series. Similar to previous studies employing periodic ridges,^23,24^ the constriction periodicity and the size of the channel between two consecutive constrictions have to be optimized to support fast cell recovery between two consecutive contact-mediated compressions. Increasing the height of the channels in the MDR-HDR might help, too, such that the cell will be in contact only with the two lateral channel walls, providing a more useful surface for the cell volume exchange. Currently, there is still no clear experimental evidence on the size of opened pores and their relationship with operating parameters, device geometry and cell physical properties. In particular cell mechanical properties such as membrane tension and underlying tension in the cell cytoskeleton can affect pore density and size. More investigation in this direction is needed to shed light on the impact of cell mechanics on mechanoporation and to enlarge the applicability of the method.

## Materials and methods

### Master fabrication

The microfluidic device was designed using KLayout software. To avoid clogging in the channels and PDMS collapse, a filter was included as an array of circular pillars (diameter = 100 μm) with periodicity of 50–200 μm after the inlet chamber. The master used for the fabrication of PDMS devices was realized through photolithography process (EVG® 620 Automated NIL System) using AZ15nXT 450 cps photoresist (MicroChemicals GmbH). A 4′′ silicon wafer was coated with the photoresist by spin coating at 850 rpm for 30 s (Laurell WS-650Hzb-23NPP-UD-3 spin coater). After coating, the wafer was exposed to UV light (550 mJ cm^−2^) through chromium photomask containing the channel geometry. After baking, the photoresist was developed using a solution of AZ 400K developer (MicroChemicals GmbH) (1 : 3 v/v, developer : distilled water) for 2 min 30 s. The height of the fabricated structures was analysed using a stylus profiler (Bruker DektakXT-A) and it was of 18 μm. The prepared master template was functionalized with 1*H*,1*H*,2*H*,2*H*-perfluorodecyltriethoxysilane (Sigma-Aldrich) in a desiccator for 12 h before using.

### Device fabrication

The structures on the master were replicated on polydimethylsiloxane (PDMS) element by replica molding process. The base (Dow Corning Sylgard® 184) and curing agent were mixed in ratio 10 : 1, degassed, poured on the master and polymerized in the oven at 75 °C for 1.5 h. The cured PDMS was then peeled off and it was punched using biopsy puncher (Imtegra GmbH) in correspondence of the inlet (I.D. 1 mm) and the outlet (I.D. 1.5 mm) chambers. The punched PDMS replica was then bonded on glass coverslip (40 × 24 mm, thickness 2, Hecht) by activating their surface using an air plasma process (50 W, 30 s, Gambetti, Tucano plasma reactor). The bonded PDMS device was incubated at 75 °C for 12 h.

### Microfluidic setup assembly

The microfluidic setup included a pressure controller (Fluigent MFCS™ EX) equipped with a channel that is able to provide maximum pressure of 7 bar, operated by a software MAESFLO™ (Fluigent, Germany). The pressure controller was connected *via* Tygon tubing (OD = 4 mm, ID = 2.5 mm) to a vial containing CO_2_-independent Leibovitz's L-15 (Gibco, Cat. no. 21083027) cell culture media (CMV) which was further connected to a flow sensor (Fluigent flow rate platform, XL) using FEP tubing (O.D. = 1.5 mm, I.D. = 500 μm, Kinesis GmbH) to fill the flow sensor with the same media. The device was filled with L-15 media from a tubing connected to the outlet to avoid any air bubbles in the system. Another FEP tubing (O.D. = 1.5 mm, I.D. = 500 μm, Kinesis GmbH) was filled with cell suspension (CST) whose one end was connected to the flow sensor (flow unit – XL, Fluigent) and the other end was fitted inside the device inlet. The cell flow through the microfluidic device was activated and monitored by increasing the applied pressure through the microfluidic pressure controller while recording in real time the corresponding flow rate measured by the in-line flow sensor.

### Cell culture

Cells were cultured according to standard mammalian tissue culture protocols and sterile techniques at 37 °C in 5% CO_2_ and tested in regular intervals for mycoplasma. HeLa K and hTERT RPE-1 cells were a kind gift from Jonathon Pines (ICR, London, UK). HeLa K H2B-mCherry/GFP-tubulin was a kind gift of Daniel Gerlich (IMBA, Vienna, Austria). For cell proliferation analysis hTERT RPE-1 FRT/TR cells expressing endogenously tagged histone 3.1-mTurquoise2^48^ and HeLa K H2B-mCherry/GFP-tubulin were used. For all other experiments hTERT RPE-1 and HeLa K cells were used.

HeLa K cells were cultured in DMEM (Gibco, Cat. no. 41966) supplemented with 10% (v/v) FBS (Gibco), 1% (v/v) Glutamax (Gibco), 1% (v/v) penicillin–streptomycin (Sigma-Aldrich) and 0.5 μg ml^−1^ amphotericin B (Sigma-Aldrich). hTERT RPE-1 and hTERT RPE-1 FRT/TR cells were cultured in DMEM/F12 (Sigma-Aldrich, Cat. no. D6421) supplemented with 10% (v/v) FBS, 1% (v/v) Glutamax, 0.26% (v/v) sodium bicarbonate (Gibco), 1% (v/v) penicillin–streptomycin and 0.5 μg ml^−1^ amphotericin B. BJ fibroblasts were cultured on plates coated with 0.1% gelatine (VWR) in Eagle's minimum essential medium (EMEM) (LGC Standards, Cat. no. ATCC 30-2003) supplemented with 10% FBS and 1% (v/v) penicillin–streptomycin. U2OS cells were cultured in DMEM (Gibco, Cat. no. 31966047) supplemented with 10% (v/v) FBS and 1% (v/v) penicillin–streptomycin (Gibco).

Before and during PM, cells were kept in CO_2_-independent Leibovitz's L-15 media (Gibco, Cat. no. 21083027) supplemented with 10% (v/v) FBS, 1% (v/v) Glutamax, 1% (v/v) penicillin–streptomycin and 0.5 μg ml^−1^ amphotericin B (in case of U2OS without amphotericin B).

Before the PM, cells were washed once with PBS and detached with Trypsin–EDTA (0.05%) (Gibco). Trypsinisation was stopped by addition of appropriate cell culture media. After spinning down cells were re-suspended in L-15 media, filtered using the CellTrics filter with the 50 μm diameter (Sysmex) and counted. In case of experiments with HeLa K 20 000 cells per condition were used (except for determination of cell viability using propidium iodide staining where 50 000 cells were used), in case of hTERT RPE-1 50 000 cells and in case of U2OS 32 000 cells were used. After the PM, cells were spun down, washed once with an appropriate cell culture media and seeded, or immediately analysed.

### Real-time deformability cytometry (RT-DC)

The RT-DC measurements were performed by using the same setup and device described in a previous publication.^49^ Briefly, the cells were trypsinised and were centrifuged at 100*g* for 5 min. The supernatant was removed, and the cells were resuspended at a concentration of around 5 × 10^6^ cells per ml in 1× PBS buffer with 0.5% (w/v) methylcellulose having viscosity of 15 mPa s. The cell samples were passed through RT-DC devices with 30 μm square channel geometry at a flow rate of 0.16 μL s^−1^. All the samples were processed at room temperature within 30 min of preparation. All analysis was done using the open source software ShapeOut.^50^ The Young's moduli of the cells were calculated using ShapeOut after filtering out the events falling out of area range of 80–750 μm^2^ and area ratio range of 1–1.05.

### Propidium iodide staining

For analysis of cell viability cells were taken right after the PM and cell suspension in L-15 media was supplemented with 1 μg ml^−1^ propidium iodide (Sigma-Aldrich) staining dead cells. The cell analyser BD LSRII (BD Biosciences) was used to detect propidium iodide (PI) positive/negative cells, up to 20 000 events were counted. Data were analysed in BD FACSDiva Software Version 8.0.2.

### Cell proliferation analysis

Cells after PM were plated in a 96 well plate (Greiner Bio-One), after 4 h cells were washed twice with PBS and imaging media (DMEM without phenol red and riboflavin – Gibco, cat. no. 041-96 205M, supplemented with 10% (v/v) FBS, 1% (v/v) Glutamax, 1% (v/v) penicillin–streptomycin and 0.5 μg ml^−1^ amphotericin B) was added. Afterwards fluorescent microscopy imaging of histone 2B and histone 3.1 was performed using ImageXpress Micro XLS wide-field screening microscope (Molecular Devices) equipped with 10×, 0.5 numerical aperture Plan Apo air objective (Nikon). After 24 h cells were imaged again. Image analysis for counting number of nuclei based on histone 2B and histone 3.1 was done in MetaXpress software (Molecular Devices). The ratio between number of nuclei 24 h after PM and number of nuclei in time point 0 was calculated and it is called a fold increase in cell number.

### FITC-dextran delivery

4 kDa FITC-dextran and 70 kDa FITC-dextran (Sigma-Aldrich) were used as model cargo. 4 kDa and 70 kDa FITC-dextran were dissolved in water and were used in the final concentration of 0.2 mg ml^−1^. FITC-dextran was added to cell suspension right before flowing it through the device without any pre-incubation. To control delivery independent of PM, cell suspension was mixed with FITC-dextran and incubated for the same time as treated samples (Ctrl + FITC-dextran). The whole process of PM was done at room temperature within an hour. Cells after PM were plated in a 96 well plate and after 20 h cells were washed once with PBS, trypsinised and analysed. FITC-positive cells were detected using the cell analyser FACS Caliber™ (BD Biosciences) or BD FACSCanto II SORP (BD Biosciences), up to 5000 total events were counted. To distinguish FITC positive cells due to delivery *via* PM, we excluded cell counts resulting from autofluorescence, endocytosis and surface binding (Ctrl + FITC-dextran) (Fig. S3†). The gating strategy for FITC positive cells was defined that less than 1% of Ctrl + FITC-dextran cells were classified as FITC positive. The threshold for FITC positive cells was individually determined for each experiment respecting the gating strategy because contribution of delivery independent of PM as well as cell autofluorescence can vary between different experiments. Data were analysed in BD CellQuestTM or BD FACSDiva Software Version 9.0.

### Formation and delivery of SpCas9NLS–sgRNA ribonucleoprotein (Cas9–sgRNA RNPs)

SpCas9NLS protein (=wild-type Cas9 nuclease from *Streptococcus pyogenes*, fused with a C-terminal nuclear localization signal (NLS) (158.4 kDa), Eupheria Biotech 5000 ng μL^−1^) was diluted to 25 μM with HEPES/KCl buffer pH 7.25 (20 mM HEPES, 150 mM KCl, 1 mM DTT). sgRNA (custom made modified single guide RNA (32.4 kDa), Synthego Corporation) was dissolved to 100 μM in Tris–EDTA-buffer pH 8.0 (Synthego Corporation) and diluted to 25 μM with nuclease-free water (Synthego Corporation).

sgRNA sequence ‘targeting GFP’: G*G*C*CACAAGUUCAGCGUGUC + Synthego modified EZ Scaffold.

sgRNA sequence ‘non-targeting’: C*G*U*ACGAUCUCGUAAACGCG + Synthego modified EZ Scaffold.

N*N*N* indicate 2′-O-methyl analogues and 3′-phosphorothioate internucleotide linkages.

Per sample 1 μL Cas9 protein dilution (25 μM) and 5 μL sgRNA ‘targeting GFP’ or ‘non-targeting’ dilution (25 μM) were combined in a micro tube to form the RNP with a molar ratio of Cas9 : gRNA = 1 : 5. The contents were mixed by flicking the tubes, briefly centrifuged and incubated 10 min at room temperature.

For each sample 6 μL RNP were pipetted into a micro tube, 19 μL U2OS cell suspension with a density of 2 million per ml were added to reach a final concentration of 1 μM RNP, then kept on ice until the PM.

Cells were treated with 40–4μm and 60–4μm (*L*_c_–*W*_c_) devices at a pressure of 3 bar. Cell suspension only and cell suspension with ‘non-targeting’-RNP were used as controls. After the PM until seeding, cell suspension was kept at room temperature. All samples were centrifuged for 10 min at 100*g*, resuspended in 100 μL of appropriate cell culture media, seeded in a 96 well cell culture plate and incubated at 37 °C. 22 h later cells were trypsinised and transferred into a 24 well cell culture plate. 90 h after the PM cells were trypsinised and the expression of GFP was analysed with the cell analyser FACS Calibur™ (BD Biosciences) counting up to 5000 total events.

In parallel, an aliquot of 15 000 cells at timepoint 22 h was seeded into a μ-slide 8 well microscopy chamber (Ibidi GmbH). 2 days later cells were incubated with DAPI (Sigma-Aldrich) 1 μg ml^−1^ in L-15 media for 20 min at 37 °C. Cells were washed twice with PBS and covered with L-15 media. Live-microscopy was performed with Deltavision inverted microscope (DV Elite Imaging system, Olympus IX-71 inverted microscope, applied precision) equipped with 10× objective (UPLSAPO, 0.4 NA, WD 3.1 mm, Olympus).

### Determination of cell diameters

To determine cell diameters, cells were detached with trypsin–EDTA (0.05%), trypsinisation was stopped by addition of appropriate cell culture media. Afterwards the media was replaced with PBS and bright-field imaging was performed using Nikon Eclipse Ti (Nikon) equipped with 20×, 0.45 numerical aperture Plan Fluor air objective (Nikon). The cell diameter was determined using Fiji software.^51^

### Determination of cell velocity

The mean cell velocity of HeLa K was calculated for each device and for each operating pressure (Table S1†) by the analysis of the cell flow inside the channel using an inverted microscope (Zeiss, Axio Observer.A1 equipped with 5×, 0.12 numerical aperture A Plan air objective). Videos were recorded at 6000 fps (EoSens CL 1362, Mikrotron) and cell position was analysed by Fiji software.^51^ Mean cell velocity was calculated as distance travelled by cell along the channel divided by the correspondent time, calculated through the ratio between the number of frames and the recording frame rate (6000 fps).

### Statistical analysis

Prism 6.0 (GraphPad) was used for statistical analysis. All applied statistical analysis and respective statistical information are indicated in respective figure legends. *P* values higher than 0.05 were considered as non-significant (ns).

## Author contributions

Conceptualization: S. G. and J. M.; methodology: A. U., R. G., S. G., J. M., M. A., F. B., J. G.; investigation: A. U., R. G., M. A., S. D. G.; data analysis and interpretation: A. U., R. G., S. D. G., M. A., S. G., J. M.; writing—original draft: A. U., R. G., S. G., J. M.; writing—review & editing: A. U., R. G., S. G., J. M., M. A., S. D. G., F. B., J. G.; funding acquisition: A. U., S. G., J. M., J. G., F. B.; supervision: S. G. and J. M.

## Funding

This work was supported by the ERC under the European Union's Horizon 2020 research and innovation program (grant agreement no. 680042; J. M., no. 742133 and 825825; F. B., no. 953121; S. G.), the DFG (MA5831/1-1 and MA5831/3-1; JM), a DIGS-BB fellowship (A. U.) and the State of Saxony and the European Regional Development Fund (S. G.).

## Conflicts of interest

The authors declare that they have no competing interests. TU Dresden and MPG has filed a patent application based on this work, in which S. G, R. G., A. U., J. M. and J. G. are listed as inventors.

## Supplementary Material

LC-021-D0LC01224F-s001

LC-021-D0LC01224F-s002
