# Supplementary material for: Efficient and gentle delivery of molecules into cells with different elasticity via Progressive Mechanoporation
Source: Lab Chip. 2021 May 12;21(12):2437–52. doi: 10.1039/d0lc01224f (PMC8204113; doi:10.1039/d0lc01224f)
Supplement: LC-021-D0LC01224F-s002 [file LC-021-D0LC01224F-s002.pdf]

Electronic Supplementary Material (ESI) for Lab on a Chip.
This journal is © The Royal Society of Chemistry 2021

Supplementary Information

Efficient and gentle delivery of molecules into cells with different elasticity via Progressive Mechanoporation

Alena Uvizl[1,5] Ruchi Goswami[2,5], Shanil Durgeshkumar Gandhi[1], Martina Augsburg[3], Frank Buchholz[3], Jochen Guck[2], Jörg Mansfeld[1,4*], Salvatore Girardo[2*]

[1]Cell Cycle, Biotechnology Center, Technische Universität Dresden, 01307 Dresden, Germany
[2]Max Planck Institute for the Science of Light & Max-Planck-Zentrum für Physik und Medizin, 91058 Erlangen, Germany
[3]Medical Systems Biology, Medical Faculty and University Hospital Carl Gustav Carus, TU Dresden, 01307 Dresden, Germany
[4]The Institute of Cancer Research, London SW7 3RP, UK
[5]These authors contributed equally: Alena Uvizl, Ruchi Goswami

*Corresponding authors: salvatore.girardo@mpl.mpg.de, jorg.mansfeld@icr.ac.uk

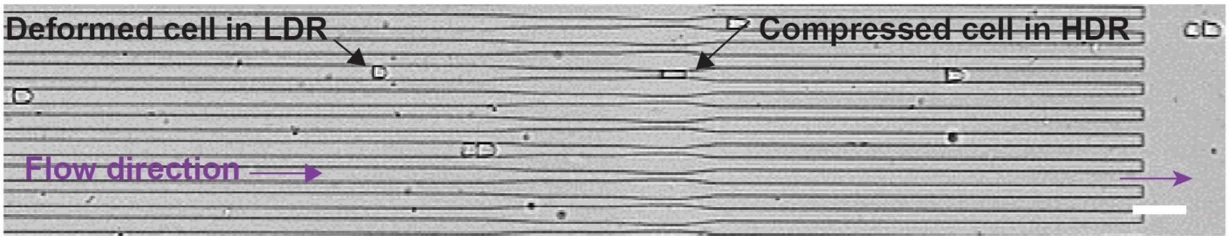

Fig. S1 Bright field image of a microfluidic device, 40µm-6µm (L_c-W_c), showing HeLa K cells flowing through 9 microfluidic channels under an applied pressure of 5 bar. The scale bar is 50 µm.

Supplementary Video

Video showing RPE-1 cells flowing through a microfluidic device 40µm-4µm (L_c-W_c) under an applied pressure of 3 bar at a frame rate of 15 fps (400x slower). The video was recorded using an inverted microscope (Zeiss, Axio Observer.A1 equipped with 5x, 0.12 numerical aperture A Plan air objective) at 6,000 fps (EoSens CL 1362, Mikrotron). Frame numbers are reported in the video.

Relevant parameters

For a fixed device geometry, the flow rate increased with the pressure. For different device geometries, the flow rate at the inlet, Q_i, varied with the device flow resistance, decreasing with the increase of flow resistance in each channel (R_{fc}), according to Hagen-Poiseuille equation, $R_{fc} = 12\mu L/\pi w h^3$, where μ is the dynamic viscosity ($N \cdot s\ m^{-2}$) and L, w and h are the length, width and height of the channel, respectively[1]. In agreement with this relation, a decrease in the flow rate with the increase of L_c and decrease in the W_c was observed. For all four different devices dimensionless Reynolds number (Re), a ratio of inertial forces to viscous forces, was calculated as $Re = 4\, Q_c/P\, \nu$, where $Q_c = Q_i/60$, is the volumetric flow rate ($m^3\ s^{-1}$), P is the perimeter of LDR channel (m) and ν is the kinematic viscosity ($m^2\ s^{-1}$). The fluid shear stress (FSS, τ) for rectangular channel with $h > w$ was calculated as $\tau = 6\,\mu\, Q_c/h\, w^2$, where h and w are the height and width of the LDR channel, respectively[2]. For comparison with previous studies the velocity of cells was measured for each operating pressure by recording a video of cells flowing through parallel channels at 6000 fps (Table S1). Mean cell velocity was calculated as distance travelled by cell along the channel divided by the correspondent time (measured as number of frames/6000 fps). For 3, 4 and 5 bar pressure, we measured average cell velocity of 452 mm s^{-1}, 693 mm s^{-1} and 945 mm s^{-1}, respectively.

Device (L_c-W_c)	Pressure (bar)	Flow rate (µL min^{-1})	Reynolds number (Re)	Fluid Shear Stress, τ (N m^{-2})	Mean cell velocity (mm s^{-1})
40µm-6µm	3	531	8.9	194.5	496
	4	818	13.7	299.5	722
	5	1,170	19.6	428.5	1012
60µm-6µm	3	482	8.1	176.4	437
	4	735	12.3	269.0	715
	5	1,021	17.1	374.0	1047
40µm-4µm	3	448	7.5	164.2	514
	4	629	10.6	230.4	703
	5	928	15.6	339.8	998
60µm-4µm	3	408	6.9	149.5	391
	4	600	10.1	219.5	670
	5	883	14.8	323.3	913

Table S1 Table of recorded and calculated flow parameters for four different devices (40µm-6µm; 60µm-6µm; 40µm-4µm; 60µm-4µm (L_c-W_c)) for three operating pressure (3, 4 and 5 bar). Mean cell velocity for Hela K cells was calculated by analysing the cell position in a microchannel over time.

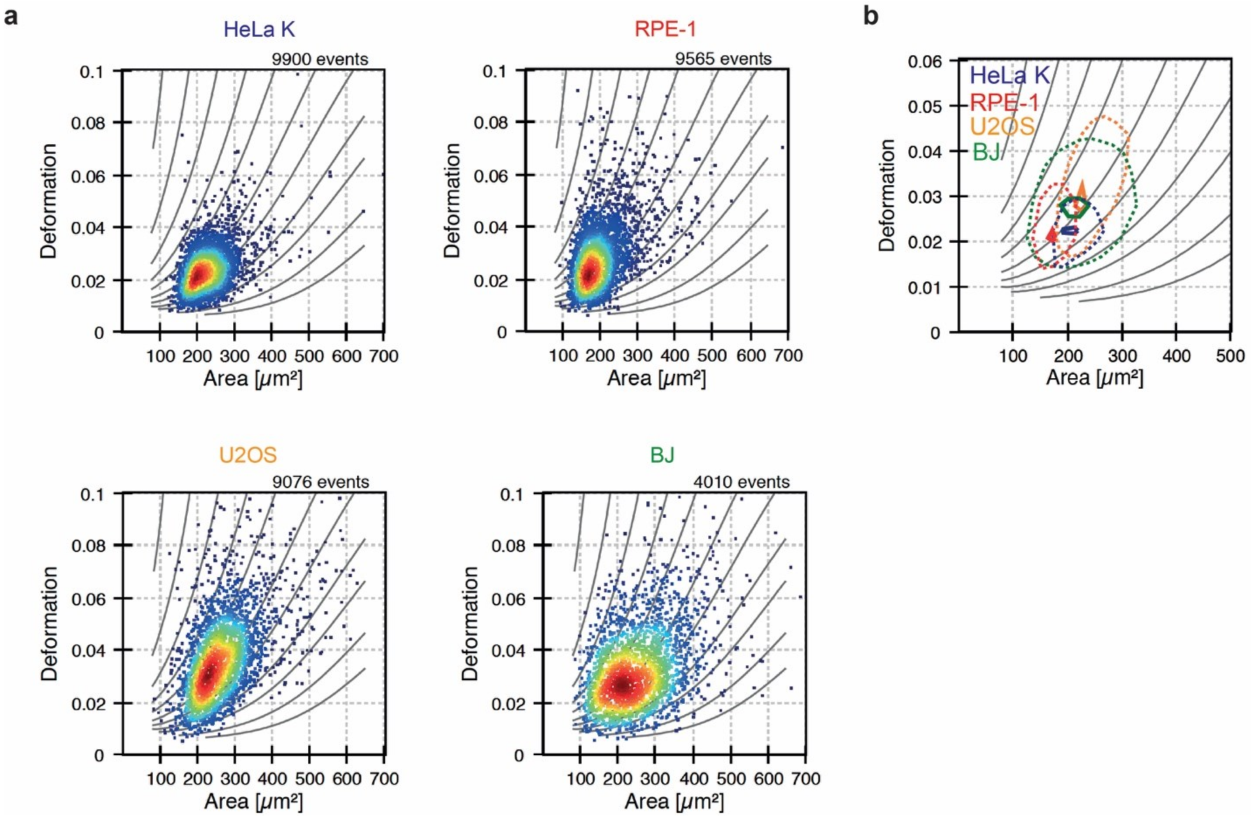

Fig. S2 (a) RT-DC measurements of individual cell types (HeLa K, RPE-1, U2OS and BJ) representing the deformation for each cell event with its corresponding measured cell area (μm^2). Total number of measured cell events is indicated. (b) Comparison of contour plot of cell deformation versus area for the four cell types (HeLa K, RPE-1, U2OS and BJ).

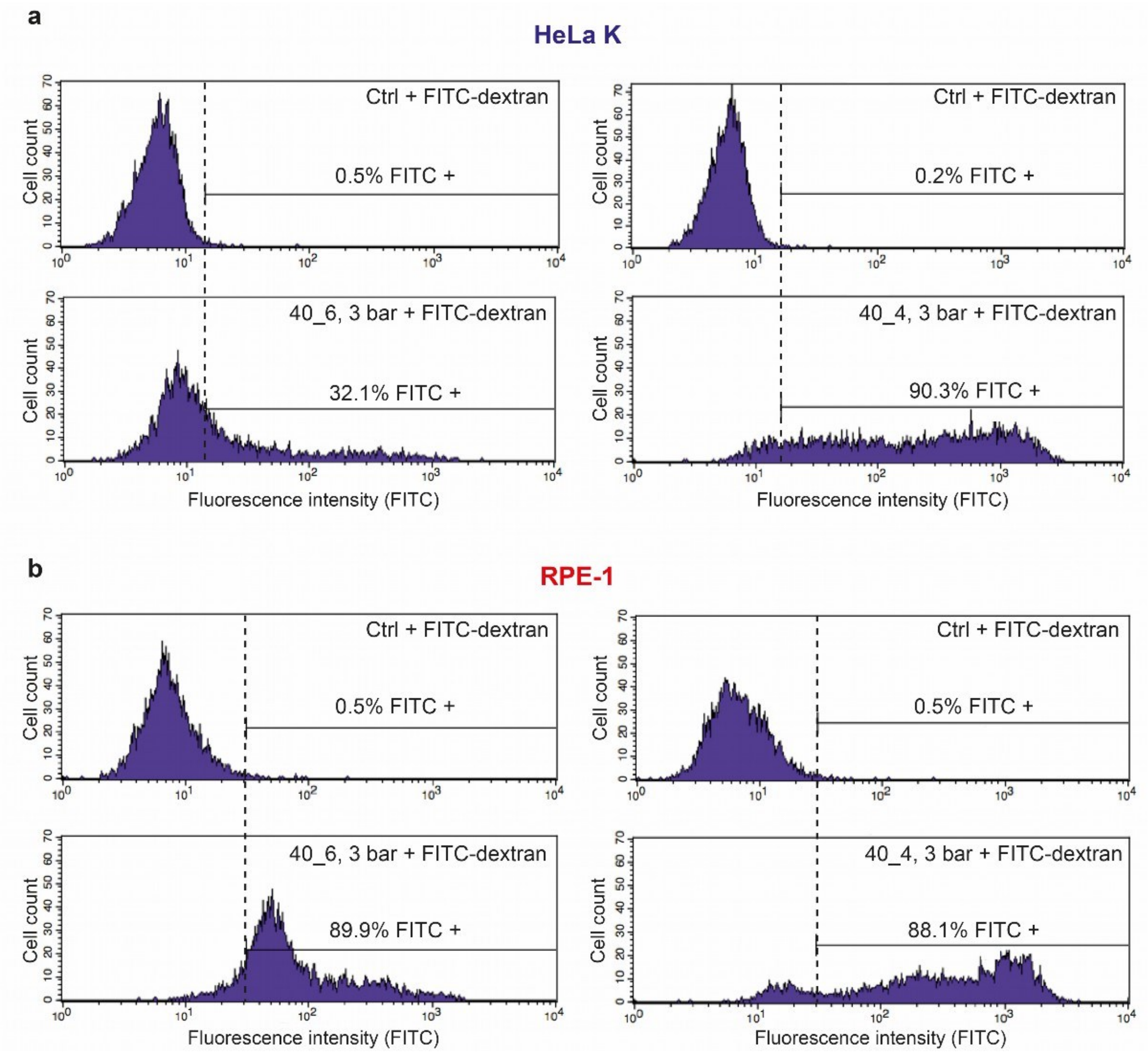

Fig. S3 Examples of fluorescence intensity histograms obtained by FACS analysis of HeLa K (a) and RPE-1 (b) cells. Cells were exposed to 4 kDa FITC-dextran without PM (Ctrl + FITC-dextran) or with PM using 40μm-6μm (L_c-W_c) and 40μm-4μm (L_c-W_c) devices under an applied pressure of 3 bar. In individual measurements of up to 5,000 cells were analysed. Cells with or without PM were exposed to the same concentration of 4 kDa FITC-dextran over the same period of time. To distinguish FITC positive cells (FITC +) due to delivery via PM, we excluded cell counts resulting from autofluorescence, endocytosis and surface binding (Ctrl + FITC-dextran). The gating strategy for FITC + cells was defined such that less than 1% of Ctrl + FITC-dextran cells were classified as FITC +.

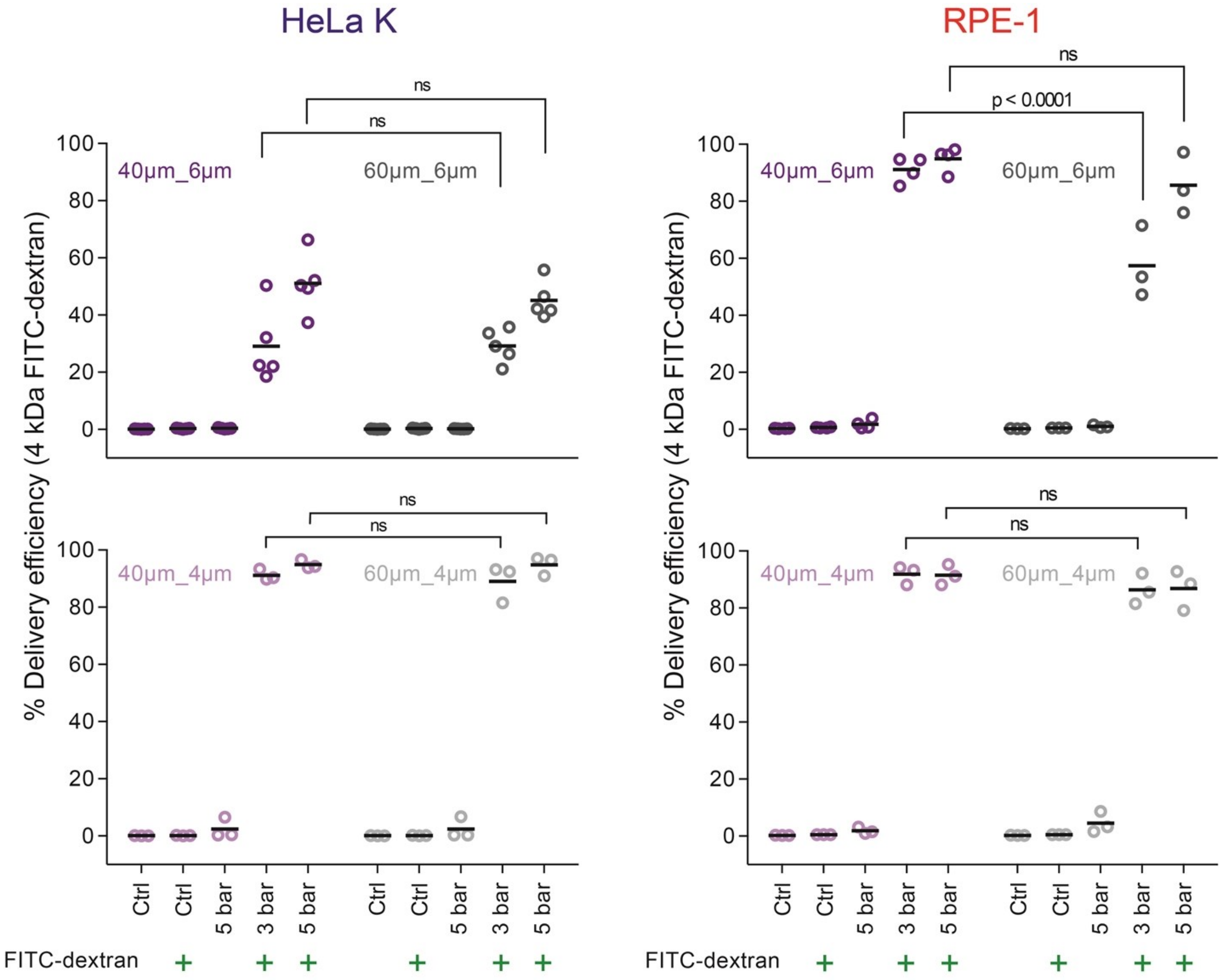

Fig S4. Comparison of delivery efficiency by progressive mechanoporation using devices with 40 µm (40µm-6µm and 40µm-4µm (L_c-W_c)) and 60 µm (60µm-6µm and 60µm-4µm (L_c-W_c)) length of constriction (L_c) in HeLa K and RPE-1 cells. The data represented in this figure are taken from Fig. 3 and Fig. 4. The delivery efficiency was measured by FACS for the following conditions: not treated cells (Ctrl); cells treated with operating pressure of 3 bar and 5 bar. The symbol (**+**) represents the addition of 4 kDa FITC-dextran to the cell suspension. Individual measurements (circles) and mean values (line) are reported. Significance according to Sidak's two-way analysis of variance (ns = not significant).

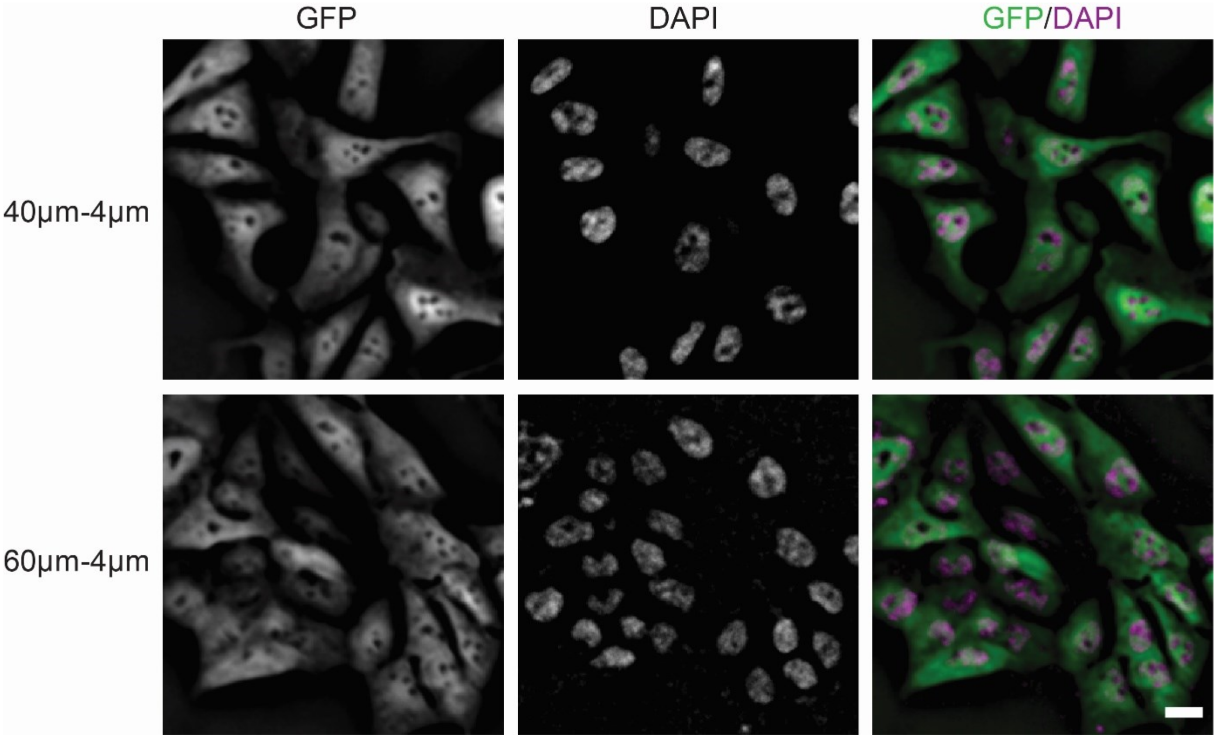

Fig. S5 Fluorescent microscopy images of U2OS cells expressing GFP after the PM without any cargo using 40μm-4μm and 60μm-4μm (L_c-W_c) devices under an applied pressure of 3 bar. Cells were processed as it is indicated in Fig. 6a. DAPI was used for DNA staining. The scale bar is 20 μm.

Bibliography

1 P. Sethu, A. Sin and M. Toner, *Lab on a Chip*, 2006, **6**, 83–89.
2 Y. Duan, N. Gotoh, Q. Yan, Z. Du, A. M. Weinstein, T. Wang and S. Weinbaum, *Proceedings of the National Academy of Sciences*, 2008, **105**, 11418-11423.
